# Effects of the Breeding Strategy Beef-on-Dairy at Animal, Farm and Sector Levels

**DOI:** 10.3390/ani13132182

**Published:** 2023-07-03

**Authors:** Rana Hamas Ahmed, Christin Schmidtmann, Julius Mugambe, Georg Thaller

**Affiliations:** Institute of Animal Breeding and Husbandry, Christian-Albrechts-University Kiel, Hermann-Rodewald-Straße 6, 24118 Kiel, Germany; cschmidtmann@tierzucht.uni-kiel.de (C.S.); jmugambe@tierzucht.uni-kiel.de (J.M.);

**Keywords:** beef on dairy, crossbreeding, calving difficulty, still-birth, farm economics

## Abstract

**Simple Summary:**

Multiple factors including decreasing milk prices and rising demand for high-quality beef have resulted in augmented utilization of beef sires in dairy herds. This breeding strategy, which is commonly referred to as Beef-on-dairy (BoD), is aimed to improve the economic performance of the farm by producing higher-valued crossbred calves with better carcass traits. Along with the positive aspects of economics, BoD can result in unwanted negative consequences including increased gestation length, dystocia and stillbirth rates resulting in compromised animal welfare. Modern breeding tools including genomic selection for the selection of beef sires with favorable calving traits and the development of specialized breeding indexes can help in overcoming the potential negative aspects associated with the BoD strategy.

**Abstract:**

The decline in farm revenue due to volatile milk prices has led to an increase in the use of beef semen in dairy herds. While this strategy (“Beef-on-dairy” (BoD)) can have economic benefits, it can also lead to unintended consequences affecting animal welfare. Semen sale trends from breeding organizations depict increasing sales of beef semen across the globe. Calves born from such breeding strategies can perform better when compared to purebred dairy calves, especially in terms of meat quality and growth traits. The Beef-on-dairy strategy can lead to unintentional negative impacts including an increase in gestation length, and increased dystocia and stillbirth rates. Studies in this regard have found the highest gestation length for Limousin crossbred calves followed by calves from the Angus breed. This increase in gestation length can lead to economic losses ranging from 3 to 5 US$ per animal for each additional day. In terms of the growth performance of crossbred animals, literature studies are inconclusive due to the vast differences in farming structure across the regions. But almost all the studies agree regarding improvement in the meat quality in terms of color, fiber type, and intra-muscular fat content for crossbred animals. Utilization of genomic selection, and development of specialized Beef-on-dairy indexes for the sires, can be a viable strategy to make selection easier for the farmers.

## 1. Introduction

The terms “Beef-on-dairy” (BoD) and “dairy beef” have gained significant interest in the literature recently. Dairy beef is an inclusive form that describes all kinds of meat produced from the dairy system, including meat from surplus dairy calves and culled cows [1], while BoD refers to the type of breeding system describing the use of beef semen in dairy herds with an aim to produce calves with higher economic value for the meat. Due to undesired meat characteristics and a lower meat-to-bone ratio [2], surplus male dairy calves usually are unwanted products of the dairy system. This results in lower market value as compared to purebred beef calves and beef crossbred calves. There are possibilities for improving beef production from dairy systems by inseminating dairy cows with fast-growing beef breeds resulting in additional economic gain [3]. Due to these benefits increased utilization of beef semen in dairy herds across the globe has been observed. The insemination data records from England regarding the sales of beef semen document increases in the sales of beef semen from 25% in 2013 to 47.6% in 2020 [4]. Similarly in North European countries such as Denmark, Sweden, and Netherlands, the proportion of beef semen used in dairy herds ranges between 20 and 25% [5]. The insemination data reported from the United States depicts a similar picture, where beef semen counted for almost 20% of inseminations in 2019 [6]. This increase in the sale of beef semen in the US market, accompanied by a decline in the sale of dairy breed semen from 23.2 million doses in 2017 to 18.3 million doses in 2020, indicates a significant increase in the utilization of beef semen in dairy herds [7,8]. Similar results have been shown by a study from the United States [9] where an eight percent increase (18.2 vs. 26.1%) in the use of beef semen on Holstein dairy herds from 2019 to 2021 was reported. Trends regarding sales of beef semen are summarized in Table 1.

Prices of beef products are increasing [10] and are anticipated to keep increasing in the near future [11]. Combined with volatile milk market prices and higher revenues from the BoD calves, a further increase in the use of beef semen in dairy herds is expected by many farms. The economic gain from BoD crossbreeding is not only limited to higher revenue for the farms, but also can create higher consumer acceptance, which is an important aspect of BoD. The studies comparing meat from pure dairy breeds with BoD crossbreds have suggested that meat from BoD crossbreds can be marketed along with meat from traditional beef breeds due to similar aesthetic and eating qualities [12]. However, the use of beef semen in dairy herds may lead to an unwanted increase in the stillbirth rate and calving difficulty, relinquishing economic advantages due to higher veterinary costs and lower animal productivity. Furthermore, the welfare aspect of animal farming has emerged as a matter of paramount importance within the livestock industry. This increased attention can be attributed to growing ethical concerns and the escalating demand from consumers for more humane farming practices. Implementing a BoD breeding strategy potentially offers remedies to several issues but requires a careful evaluation of the farm-specific situation regarding configuration, income sources and herd fertility. The use of advanced breeding techniques such as genomic selection and appropriate BoD selection indices combined with a required level of vigilance, can effectively mitigate potential difficulties such as elevated calving difficulty. This review aims to provide a comprehensive overview of the diverse impacts of the BoD breeding strategy, including its effects on animal welfare, farm economics, and the environment. In order to provide a holistic view of the BoD strategy, this review is not limited to the breeding aspects of this strategy but also covers other important aspects of animal farming such as the economic impact and animal welfare implications of breeding decisions.

## 2. Implications of Beef-on-Dairy at the Animal Level

### 2.1. Calving Difficulty and Stillbirth

One of the significantly important aspects of the BoD breeding strategy is animal welfare that is compromised by an increase in calving difficulty and stillbirth. The use of beef semen on dairy cows, especially from late-maturing beef breeds, can lead to a significant increase in the cases of calving difficulty in the herd. This increase can lead to negative consequences including a decline in farm revenue due to higher veterinary costs, loss in milk yield, and increased calving interval along with compromised animal welfare. A study on 1,598,363 calving records from Swedish dairy farms revealed a significantly higher risk of calving difficulty in dairy herds when cows were inseminated with the semen of fast-growing, late-maturing beef breeds such as Charolais and Limousin [12]. The higher probability of problematic calving when using beef semen in dairy herds might be associated with higher birth weight, the body structure of the calf, and its muscle development rate [13]. These traits differ significantly across breeds. For instance, Eriksson et al. [12] found that the difference in muscle development rates across various beef breeds significantly influences calving difficulty. Similarly, the highest growth rate along with the highest birth weight were reported for Brahman-sired crossbred calves, followed by Belgian Blue and Angus crossbreds [14]. In the same study, the crossbreds from the Brahman breed had a significantly higher birth weight accompanied by the highest incidence rate of calving difficulty.

Breed differences for problematic calving in BoD calves have also been reported by a Spanish study on 525,535 calving records [15]. The highest rate of calving difficulty was found for Holstein × Belgian Blue crossbreds followed by crossbreds of the Limousin breed. In literature, other factors including the sex of the calf and gestation length have been linked with calving difficulty in crossbreds [15,16]. Another important trait that might influence calving difficulty in the animals is the cow’s parity number as found by a study on Irish dairy farms using 2,733,524 insemination records [17]. The calving difficulty was found to be 1.39% for dairy sires and 1.35% for beef sires in first-parity animals. With increasing parity number, however, calving difficulty rose significantly reaching up to 4.11% when inseminated with beef sires in contrast to 1.83% when inseminated with dairy sires, along with an increase in gestation length [17]. No explanation was available for these results, but extended gestation length along with the selection of beef sires might have played some role in higher incidences of calving difficulty. As suggested by Berry et al. [18], variation among the beef breed sires exist, and identification of the sires which can meet the requirements for the individual farmers is of critical importance for the success of the BoD breeding strategy. Moreover, the calving difficulty seems to vary across different breeds, and within the breed, individual sires seem to have a significant influence on the trait. Most studies agree on the difference between early-maturing and late-maturing breeds, as calving difficulty is typically more often reported in late-maturing breeds. Causes of stillbirth are a little more complicated to explain in comparison to difficult calving, where feto-pelvic incompatibility is the most important factor [19]. The study by Eriksson et al. [12] found a significantly lower stillbirth rate for crossbred calves from Hereford, Limousin, Simmental, and Charolais sires compared to purebred Holstein calves. According to the authors’ opinion, it might be related to the use of early-maturing beef breed sires like Angus in dairy herds. The stillbirth rate varies significantly across various breeds; however, little information is available regarding stillbirth rates in crossbred calves. In a study on Irish herds [20], the problem of stillbirth events was found to be significantly localized on some farms, while most of the farms did not have any problems with stillbirth. Farmers with dairy breed semen reported 0.78% higher calf mortality as compared to beef semen. This might be due to the use of semen from a particular beef breed but might be also due to management, e.g., insemination of second or third parity animals and calving management. The stillbirth rate does not appear to relate to calving difficulty. This might be due to the lack of proper reporting mechanisms and optimum vigilance at the farm level. Lack of accurate data recording combined with the distribution of data due to the frequent use of selected breeds, make the comparison a challenging task. Conclusions regarding stillbirth rate becomes even more difficult by the limitation of selective sires within the breed.

Traditionally, breeding goals for the selection of sires for dairy herds have been focused on milk production and functionality traits [21], but with increasing beef and dairy crossbreeding, the development of a BoD index for the selection of beef sires to be used in the dairy herd has gained significant interest. In Ireland, BoD index ranks breeding bulls based on economic output from the calves, with the highest relative emphasis on calving difficulty and carcass characteristics [21,22]. Similarly, the Scandinavian countries Denmark, Sweden and Finland have introduced the Nordic beef-on-dairy Index (NBDI), which includes seven traits such as calving difficulty, stillbirth and carcass traits [23]. Likewise, the highest economic impact in the NBDI is attributed to calving difficulty due to its adverse impact on the dam, resulting in reduced milk yield and health complications due to dystocia, along with potential loss of revenue from the calf [24,25].

Another approach that can assist in the BoD breeding strategy is genomic selection. By using genomic selection [26], dense marker maps can be used for the prediction of breeding values [27] for beef sires and desired traits. By doing so, the selection of beef sires that cause lower calving difficulty or superior carcass quality is possible. Compared to traditional pedigree indexes, genomic selection has higher accuracy for most of the traits [28]. However, major hurdles in the implementation of genomic selection for BoD are the availability of adequate phenotypes and overall lower prediction accuracy with a combined crossbred reference population [29,30]. The latter fact is particularly important in the BoD context, as significant differences in the performance of purebred and crossbred animals exist [31]. Moreover, estimated SNP effects are usually breed-specific, which means that SNP effects predicted for one breed cannot be transferred to another breed. At this point, however, appropriate statistical methods such as the consideration of breed-specific effects in the prediction models can help [32]. 

### 2.2. Growth Traits and Meat Quality

The performance of crossbred animals is usually considered better as compared to the purebred parental generation. An Irish study on 48 male calves, fattened under three different finishing strategies, found a slightly higher growth rate of Belgian Blue crossbred calves compared to Limousin crossbreds (1.076 vs. 1.009 kg/d), but the role of finishing strategy with highest concentrate in feed was more evident [33]. These results vary slightly from those reported in the United States, where in an effort to determine the growth curve of 516 animals kept under similar nutritional management, the weight of Belgian Blue crossbreds at the age of 48 weeks was found to be significantly lower as compared to Angus and Hereford crossbreds [34]. It is difficult to conclude differences exist in the growth rate of crossbreds across different studies due to the variation in the age of slaughter and rearing of animals under different production systems. Generally, it is assumed that crossbreds of BoD animals can perform better in terms of average daily gain and final slaughter weight, but studies are inconclusive in this regard. For instance, a recent comparison of Spanish beef production by Sánchez et al. [35] on 120 animals, kept under three production systems, found no difference in the production traits of Limousin, Charolais and Holstein crossbreds. Similar results were reported from New Zealand, where the comparison of 326 BoD crossbred calves under the grazing system did not reveal any significant impact of individual sires on beef quality traits of the calves [36]. The role of production system under which calves are fattened is also of significant interest as suggested by Bittante et al. [37]. In a study on 231 calves, fattened under three different production systems, researchers found a significant influence of the production system on the carcass quality, where meat produced from BoD calves born and fattened at dairy farms had better quality in terms of tenderness and cooking losses when compared to specialized fattening farms [37]. 

The increasing demand for high-quality beef products has put significant pressure on farmers to produce meat with higher quality. The price determination system for beef relies on the quality aspects including fat contents of meat, muscle-to-bone ratio, and color of the product [33]. Along with that, the texture of the meat and flavor significantly influence the acceptance of the product by the consumers. The flavor from the BoD crossbreds was more butter and fat-like as compared to beef breeds [38]. The steak quality of BoD crossbreds was also found to be intermediate between dairy (lowest) and beef (highest) breeds. As major grading criteria for meat are based on fat and muscle contents, BoD animals were found to have more muscle as compared to dairy animals and less fat as compared to traditional beef breeds. In summary, BoD animals produced a slightly less marketable meat quantity as compared to beef breeds but were significantly higher in comparison to dairy animals [38]. Selection of the sire with a focus on marketable traits is of significant importance in this regard as found by Martín et al. [39]. A significant influence of beef sire on rib fat depth was found, and the authors concluded that the use of beef breeds on dairy animals can significantly improve the quality of desired cuts ultimately resulting in more economic gains. The heritability of meat quality traits differs significantly across various breeds. During a crossbred study on 766 Hereford cows, sired with seven different beef breed sires, it was estimated to be low for marbling (18 ± 7)% and moderate for carcass weight (36 ± 8)% and fat color (33 ± 8)%. After the exclusion of Belgian Blue and Limousin breeds from the study, heritability for the marbling trait increased significantly [40]. In this regard to some extent, it might be possible to select sires with higher carcass quality traits to use in dairy herds to improve the characteristics of the meat. 

## 3. Implications of Beef-on-Dairy at the Farm Level

### 3.1. Effect on Farm Economics

Revenues of a dairy farm originate from multiple sources of income including milk sales, the sale of surplus calves, and the slaughter value of cows. Due to volatile milk prices and low prices for purebred dairy calves, crossbreeding with beef breeds may positively influence farm profit because of the higher economic values of crossbred calves. As reported in an Italian study [41] based on 96,458 calves recorded from 2003 to 2007, Belgian Blue crossbred calves always fetched higher prices and had higher body weight at selling when compared to dairy breed calves. However, whether the BoD strategy is economically beneficial, highly depends on farm structure, stocking density, fertility rate, and the use of sexed semen [42]. At herd level, utilization of sex-sorted semen along with the use of genomic testing can provide extra revenues by decreasing the number of surplus dairy calves and replacing them with higher-valued beef crossbreds. Usually, simulations are conducted to quantify the impact of different breeding strategies on farm profitability. For instance, Hietala et al. [43] demonstrated in their simulation study that the reduction in replacement rate of heifers due the sexed semen and increased lifetime milk production, resulted in improved economic revenues because the number of non-productive heifers can be reduced, while the average milk yield per animal increased due to the higher proportion of multiparous cows in the herd. Moreover, genetic gain improved, due to the combination of sexed semen and increased selection intensity of female animals with higher genetic merits. The study also revealed that the combination of beef semen and sexed semen can be the most efficient breeding strategy to increase beef production, but not the most profitable approach [43]. The reason might be the absence of significant differences in the prices of male and female calves during the period. In contrast, a similar simulation study by Ettema et al. [44] suggested the combined use of beef and sexed semen in a dairy herd to maximize profit instead of conventional semen. Recent findings from Cabrera et al. [45] suggested that the profitability of the BoD strategy is largely influenced by the reproductive performance of the dairy herd. Utilization of sex-sorted semen in cows with higher genetic value to produce a replacement herd in the combination with beef semen to maximize the sale value of the surplus calves can be the most suitable strategy [42].

### 3.2. Effect on the Gestation Period and Conception Rate of Cows 

The conception rate of the herd and gestation length are important parameters influencing the profitability of a dairy farm. With the adaptation of the BoD breeding strategy on the farm, a slight increase in the gestation length is expected [45]. The gestation period for calves sired by the breed Angus was 1.6 days longer (276.5 d) as compared to purebred Holstein calves (274.9 d) [46]. These results are in agreement with the study by Fouz et al. [15], where Holstein purebreds had the shortest gestation period (279.1 d), followed by Belgian Blue (281.4 d). By contrast, utilization of Limousin sires resulted in a significantly higher gestation length (285 d) (Table 2). The effect of the sire breed on the gestation length of the dam was also confirmed by Reynolds et al. [47]. In their study, the average reported gestation length for Angus sired calves was 284.6 days, while it was 288.5 days for Simmental sired calves [48]. Similar results were reported from a study in New Zealand, where the average gestation length for Angus sired calves was found to be significantly shorter when compared to Hereford sired calves [16]. This study also revealed a significant effect of individual sires on gestation length of the dam. Calves from shorter gestation lengths are associated with lower calving weights, which can result in reduced calving difficulty [48,49]. Thus, selection of individual sires based on their genetic merit for gestation length can help in the overall reduction in calving problems [16].

An increase in gestation length can potentially lead to economic losses. A study on the US production system estimated that an increase in gestation length results in economic losses of 3.2 to 5.1 US$ per cow and day [51]. In comparison, Groenendaal et al. [47] estimated economic losses that ranged from 0 to 3 US $ per day depending on farm circumstances. Factors that influence these costs might include loss of milk yield, increase in the calving interval and loss of overall productivity on the farm with a longer gestation period. In general, beef semen prices are slightly higher as compared to conventional dairy semen, but prices of sex-sorted semen are almost double as compared to conventional semen [45]. However, semen prices vary significantly across countries. When considering the lower conception rate of sexed semen along with higher prices, the profit of utilization of sexed beef semen will heavily rely on the breeding strategy of the farm [42]. Regarding the difference between the conception rate of beef and dairy semen, different studies have found contradictory results. A US study on 268,174 insemination records by McWhorter et al. [8] found that the conception rate was slightly lower for Angus sires as compared to Holstein sires (33.77 vs. 34.29)% for heifers and (52.96 vs. 55.34)% for multiparous animals [8]. These findings are different from a study by Berry et al. [52], who reported higher fertility for Belgian Blue bulls compared to Angus and Holstein breeds. These differences might be attributed to the quality of semen and processing techniques as described by [53]. The fertility rate of dairy and beef semen is assumed to be at same level, Cabrera [45], but lower abortion rates have been reported by the use of beef semen [54]. This might be the reason for the use of beef semen in cows with reproductive problems, resulting in an overall lower conception rate for beef semen inseminations. 

Along with the direct economic losses due to calving problems, improved animal care can also lead to improved performance [55]. Higher calving difficulty in the dams has been associated with compromised milk production, and a decline in the fertility of the cow [56,57]. Though long-term effects of calving difficulty on the performance of the calves are not pronounced, a study from UK has found a slight decline in the milk production in the early lactation period [56].

## 4. Implications of Beef-on-Dairy on Sector Level

### Effect of Beef-on-Dairy on the Environment

Regarding the livestock sector, beef farming is considered to have the highest CO_2_ emissions and land use change among livestock production [58,59]. With the increase in global population combined with higher purchasing power of the population, demand for meat is increasing [60]. By efficiently utilizing existing resources and animal farming infrastructure to maximize the output in terms of meat, it may be possible to decrease these impacts to some extent. For instance, due to the higher growth rate of BoD crossbred calves, the optimum slaughter weight could be achieved in less time, resulting in significantly lower enteric methane emissions and ultimately leading to lower emissions associated with per kg of meat [61]. A simulation study by van Selm et al. [62], which investigated the global warming potential of New Zealand, showed that the calves born from the dairy beef production system had 29% lower emissions as compared to suckler animals. Based on per kg carcass weight, dairy beef production system had almost 22% fewer emissions (16.6 vs. 23.4 kg CO_2_ per/kg carcass). The distribution of total emissions into two categories, “birth to weaning” and “weaning to slaughter” might have resulted in overall lower emissions in this study. Another important aspect resulting in overall lower emissions from BoD might be the slightly higher growth rate of crossbred calves, thus, reaching final slaughter weight faster [63]. These results are similar to those of a Finnish study. Hietala et al. [43] compared different breeding strategies in order to find the optimum one to maximize beef production. They found that using Y-sorted sperm with the crossbreeding rate of 80% of mature herd can result in a maximum decline in greenhouse gas emissions [43]. The use of early-maturing and late-maturing beef breeds for BoD can also significantly affect the amount of environmentally harmful emissions, as more efficient animals with higher weight gain will require less days at farm, leading to lower methane footprint per kg of meat [64]. A comparative study of different meat production systems by Nguyen et al. [10] found that the highest emissions per kg of meat were associated with the suckler calf system. The longer the calves remained on the farm, the higher the associated emissions. With the increased efficiency of milk production resulting in decreased requirement of replacement heifers, there is a possibility that the majority of the beef production might shift to the suckler system. To minimize the impact of such a farming style, crossbreeding dairy herds with beef semen in the conjugation of sexed semen might be a sustainable approach for beef farming.

## 5. Conclusions and Recommendations

The major driving force behind increasing BoD breeding is higher revenue due to the economically more valuable calves. Despite the increasing popularity of BoD crossbreeding due to its potential economic benefits, such breeding approaches require careful evaluation of the farm structure and herd reproductive performance to minimize potential risks. The BoD breeding strategy can result in increased calving difficulty, compromising profitability due to increased veterinary costs and loss of milk yield. In this regard, the role of individual sires rather than breed and parity of the dams appears to be most important. 

Alongside the economic aspect, the farming industry is facing immense pressure from policy makers and society due to the environmental footprint of agriculture. The share of beef farming in these emissions is significantly higher compared to dairy farming based on produced product unit [65], but utilization of a well-planned BoD strategy can indirectly reduce these impacts by replacing surplus dairy calves with BoD calves having higher growth potential and market prices [66,67]. An unpublished model study from Texas Tech University suggested a possible reduction in the carbon footprint of meat by up to 50% with the BoD strategy [68], but these estimates are extraordinarily high when compared to the reported studies from New Zealand 22% [62], and Finland 29% [43], respectively. Utilization of beef semen is expected to keep increasing in the near future despite some negative influence on animal welfare. The development of specialized selection indices for beef sires based on breeding values for traits of interest including calving ease, calf survival, growth rate, carcass weight and marbling quality can be the most applicable approach in this regard.

## Figures and Tables

**Table 1:** Trends regarding sales of beef semen across globe.

Country	Year	Sale (%)	Reference
England	2013	25	AHDB ^1^ (2020) [4]
	2020	47.6	
Denmark	2021	30	DAFC ^2^ (2021) [5]
Netherland	2020	20	DAFC (2021) [5]
Sweden		20	DAFC (2021) [5]
United States	2019	20	Wen Li (2019) [6]
	2021	26.1	Lauber, M. R (2023) [9]

^1^ AHDB = Agriculture and Horticulture Development Board. ^2^ DAFC = Danish Agriculture & Food Council.

**Table 2:** Reported gestation length of dams.

Breed	Gestation	No of Animals	Reference
Hol ^1^ × Hol	274.9	107	Scanavez, A. L. (2019) [46]
Hol × Hol	279.1	457,070	Fouz, R. (2013) [15]
Hol × Hol	279		ICBF (2020) [50]
Ang × Ang	283		ICBF (2020) [50]
Hol × Ang ^2^	276.5	107	Scanavez, A. L. (2019) [46]
Hol × Wbb ^3^	281.4	32,174	Fouz, R. (2013) [15]
Hol × Lim ^4^	285	43,348	Fouz, R. (2013) [15]

^1^ Hol = Holstein, Ang ^2^ = Angus, Wbb ^3^ = Belgian Blue, Lim ^4^ = Limousin. ICBF = Irish Cattle Breeding Federation [50].

## Data Availability

Not applicable.
